# Macrophage–sensory neuronal interaction in HIV-1 gp120-induced neurotoxicity^‡^

**DOI:** 10.1093/bja/aeu311

**Published:** 2014-09-16

**Authors:** P. J. Moss, W. Huang, J. Dawes, K. Okuse, S. B. McMahon, A. S. C. Rice

**Affiliations:** 1 Pain Research Group, Department of Surgery and Cancer, Faculty of Medicine and; 2 Department of Life Sciences, Faculty of Natural Sciences, Imperial College London, London, UK; 3 Wolfson Centre for Age Related Disease, King's College London, London, UK; 4 Pain Medicine, Chelsea and Westminster Hospital NHS Foundation Trust, London, UK; 5 Current address: Institute of Medical Sciences, University of Aberdeen, Foresterhill, Aberdeen AB25 2ZD, UK; 6 Current address: The Nuffield Department of Clinical Neurosciences, Medical Sciences Division, University of Oxford, Oxford, UK.

**Keywords:** cytokines; HIV envelope protein; macrophages; maraviroc; peripheral nervous system diseases, peripheral neuropathies

## Abstract

**Background:**

Human immunodeficiency virus (HIV)-associated sensory neuropathy (SN) is the most frequent neurological complication of HIV disease. Among the probable mechanisms underlying HIV-SN are neurotoxicity induced by the HIV glycoprotein gp120 and antiretroviral therapies (ART). Since HIV-SN prevalence remains high in patients who have not been exposed to toxic ART drugs, here we focused on gp120-mediated mechanisms underlying HIV-SN.

**Methods:**

We hypothesized that a direct gp120–sensory neurone interaction is not the cause of neurite degeneration; rather, an indirect interaction of gp120 with sensory neurones involving macrophages underlies axonal degeneration. Rat dorsal root ganglion (DRG) cultures were used to assess gp120 neurotoxicity. Rat bone marrow-derived macrophage (BMDM) cultures and qPCR array were used to assess gp120-associated gene expression changes.

**Results:**

gp120 induced significant, but latent onset, neurite degeneration until 24 h after application. gp120–neurone interaction occurred within 1 h of application in <10% of DRG neurones, despite neurite degeneration having a global effect. Application of culture media from gp120-exposed BMDMs induced a significant reduction in DRG neurite outgrowth. Furthermore, gp120 significantly increased the expression of 25 cytokine-related genes in primary BMDMs, some of which have been implicated in other painful polyneuropathies. The C–C chemokine receptor type 5 (CCR5) antagonist, maraviroc, concentration-dependently inhibited gp120-induced tumour necrosis factor-α gene expression, indicating that these effects occurred via gp120 activation of CCR5.

**Conclusions:**

Our findings highlight macrophages in the pathogenesis of HIV-SN and upstream modulation of macrophage response as a promising therapeutic strategy.

**Editor's key points**

- The mechanisms underlying human immunodeficiency virus (HIV)-associated sensory neuropathy are unclear.
- HIV glycoprotein gp120 induced delayed neurite degeneration in cultured rat dorsal root ganglion cells.
- This effect was mediated by factors released from macrophages, and was blocked by the C–C chemokine receptor type 5 (CCR5) antagonist maraviroc.
- Indirect neurotoxicity involving a gp120–CCR5 interaction in macrophages provides a plausible mechanism for HIV-induced neuropathy.

Human immunodeficiency virus (HIV)-associated sensory neuropathy (SN) is the most frequent neurological manifestation of HIV disease. It is seen in ∼40% of patients whose HIV infection is otherwise well controlled by antiretroviral therapies (ART), and is frequently complicated by intractable neuropathic pain.^1,2^ There are two major mechanisms proposed for HIV-SN: neurotoxicity induced either by the HIV-1 envelope glycoprotein gp120^3^ or by certain ART drugs.^4,5^ While certain ART drugs^4,5^ are undoubtedly neurotoxic, HIV-SN prevalence is not lower in patients who have never been exposed to these drugs,^6^ suggesting that alternative or additional factors underlie HIV-SN.

gp120 neurotoxicity is thought to result from its binding to CCR5 (C–C chemokine receptor type 5), CXCR4 (C–X–C chemokine receptor type 4), or both.^7^ Schwann cells have been suggested to play a role in the pathogenesis of HIV-SN,^8^ but there is no convincing evidence to suggest that they are direct targets of HIV infection. The majority of HIV strains, known as M-tropic viruses, preferentially target macrophages via CCR5.^9^ Growing evidence suggests that macrophages may play a role in the pathogenesis of HIV-SN. Infiltrated macrophages were found in the dorsal root ganglia (DRG) of AIDS patients with a history of HIV-SN.^10^ Simian immunodeficiency virus (SIV) only infects macrophages, not neurones, in the primate DRG, mirroring the process observed in humans^10,11^ and contradicting the theory of direct gp120 neurotoxicity. Macrophage activation by SIV precedes altered C-fibre conduction, suggesting that macrophage-mediated damage is the initiating event in HIV-SN.^11^ In rats, after treatment with HIV-1 gp120, the number of macrophages is significantly increased in the ipsilateral DRG and at the site of perineural application at the peak of mechanical hypersensitivity.^3^ When exposed to supernatant from macrophages infected with the M-tropic HIV-1_Bal_ strain, cultured DRG neurones undergo axonal degeneration.^10^ Macrophages are capable of producing more than 100 different cytokines, chemokines, and metabolites, many of which could play an unknown but crucial role in gp120-mediated pathogenesis in HIV-SN. However, this has remained unexplored.

The primary aim of this study was to elucidate further the mechanism of gp120 neurotoxicity using *in vitro* techniques. We hypothesized that: (i) gp120–sensory neurone interaction is not the direct cause of neurite degeneration; (ii) gp120 causes the release of neurotoxic mediators from peripheral macrophages that consequently lead to neurite degeneration; and (iii) gp120 induces a pro-inflammatory profile of gene expression in macrophages via CCR5.

## Methods

### Reagents

Treatment compounds, culture medium components, and the rationale for using monomeric gp120_MN_ and gp120_Bal_ are detailed in Supplementary material.

### Primary adult DRG culture

All experiments were approved by the Animal Welfare and Ethical Review Committee of Imperial College London and carried out accordingly to ARRIVE guidelines. Mixed dorsal root neuronal–glial cell cultures were obtained from adult female Wistar rats (150–250 g) using a previously published protocol.^12^ A total of 2000 –3000 cells were plated onto poly-l-lysine- and laminin-coated 16 mm coverslips in 500 µl complete-Dulbecco's Modified Eagle Medium (C-DMEM). Cultures were maintained at 37°C in a humidified environment containing 5% CO_2_, and tested after 1 day *in vitro* (DIV, 24 h). For experiments involving conditioned macrophage media and tumour necrosis factor (TNF)-α application, dissociated DRG cells were spun through an 11% bovine serum albumin cushion before plating, to reduce the presence of non-neuronal cells. Methodological details for assessing gp120-associated neurite degeneration are available in Supplementary material.

### Primary adult bone marrow-derived macrophage culture and treatment

Primary adult bone marrow-derived macrophages (BMDMs) were obtained from femurs and tibias of adult female Wistar rats (150–250 g) using a previously published protocol.^13^ Collected cells were resuspended in 50 ml macrophage complete medium (C-IMDM), which comprised Iscove's Modified Dulbecco's Medium (IMDM), 10% fetal bovine serum and Penicillin/Streptomycin antibodies and was further supplemented with macrophage colony stimulating factor, then plated across 5×100 mm culture plates. Cells were maintained at 37°C with complete-Iscove's Modified Dulbecco's Medium (C-IMDM) media changed after 2.5 h and 3 DIV. After 7 DIV, differentiated macrophages were isolated and plated at 3×10^5^ cells per 35 mm culture dish in 1 ml C-IMDM. Cells were allowed to settle at 37°C for 24 h, then media was replaced with 1 ml serum-free IMDM for 24 h before testing. BMDM cultures were treated after 9 DIV with vehicle, denatured-gp120_Bal_ or gp120_Bal_ for 4 h at 37°C. In experiments assessing CCR5 involvement, BMDMs were first preincubated in the absence or presence of the CCR5 antagonist maraviroc (1, 10, or 100 nM) for 1 h before experimentation. Cultures were then supplemented with treatment media to obtain vehicle, 1 nM denatured-gp120_Bal_ or 1 nM gp120_Bal_ in 1 ml IMDM that matched pretreatment conditions. Depending on the experiment, either mRNA was extracted to assess gene expression or cell-free supernatant was obtained for application to 1-day-old DRG cultures. More details are available in Supplementary material.

### Treatment with conditioned macrophage media or TNF-α

Media of 1-day-old DRG cultures were removed and replaced with 500 µl of the cell-free supernatant from vehicle-, denatured-gp120_Bal_, or gp120_Bal_-treated macrophage cultures, and incubated at 37°C. After 24 h, DRG cultures were fixed and immunostained with βIII-tubulin and Hoechst 32258. In a separate experiment, 1-day-old DRG cultures were treated with vehicle (phosphate-buffered saline in DMEM) or TNF-α (0.25, 0.5, 1, or 2 nM in DMEM) for 24 h, and then processed for immunocytochemistry.

### Temporal assessment of neurite outgrowth

Untreated 1-day-old DRG cultures were fixed at the start of the experiment (*t*=0 h) to establish baseline neurite outgrowth, and remaining cultures were treated with either vehicle or 2 nM gp120_MN_. After 2, 4, 8, 16, 24, or 48 h, cultures were washed then fixed with 4% paraformaldehyde. In another experiment, untreated DRG cultures were again fixed at *t*=0 h to determine basal neurite outgrowth, after which 2 nM B-gp120_MN_ was applied to the remaining cultures. Treated cultures were washed then fixed 1, 2, 3.5, 7, 10, or 24 h post-treatment.

Methods for immunocytochemistry, neurite analysis, cDNA synthesis, reverse transcription, and qPCR are described in Supplementary material.

### Cytokine microarray

BMDM cultures were established as described above. Two biological replicates were performed in two independent studies per treatment. RNA integrity was confirmed using the Eukaryote Total RNA Nano 6000 assay run with an Agilent 2100 Bioanalyser (Agilent, UK). Reverse transcription was performed using 200 ng RNA and random hexamers. Samples of cDNA were mixed with DNAse-free water to a final volume of 100 µl then mixed 1:1 with 2× TaqMan^®^ PCR Master mix (Applied Biosystems, UK) to give a final cDNA concentration of 1 ng µl^−1^. One hundred nanograms of cDNA samples (four per treatment) were loaded in biological duplicate, in a randomized order, onto three Applied Biosystems 384-well TaqMan^®^ microfluidic custom-made array cards. These cards were designed using the Applied Biosystems website (www.appliedbiosystems.com) and measured the expression of 92 different inflammatory mediators, mainly cytokines and chemokines.^14^ Relative mRNA expression was calculated using the ΔΔCt method and changes shown as fold change (treatment/vehicle). Analysis was carried out using the ReadqPCR and NormqPCR R packages.^15^ GAPDH (ID: Gapdh.Rn99999916_s1), β-actin (ID: Actb.Rn00667869_m1), Hprt1 (hypoxanthine phosphoribosyl transferase 1, ID: Hprt1.Rn01527840_m1), and X18S (Eukaryotic 18S rRNA; ID: X18S.Hs99999901_s1) were used as housekeeping genes.

### Statistical analysis

Data are presented as mean [standard deviation (sd)]. Unpaired Student's *t*-tests and one- and two-way analysis of variances (anovas) (SigmaStat 3.5, Systat Software, Inc., Germany) were performed where applicable. The mean (sd) values were calculated from identical biological replicates (independent experiments using identical experimental conditions). Each biological replicate was made up of at least two technical replicates (within treatment samples). For qPCR array cards, statistical significance was calculated by *t*-tests in R (two-sided, Welch's *t* test) on the ΔCt values. We adjusted the *P*-values using the false discovery rate correction.^15^

## Results

### gp120-associated neurite degeneration: temporal course and correlation with gp120–neuronal binding

**Fig 1:**
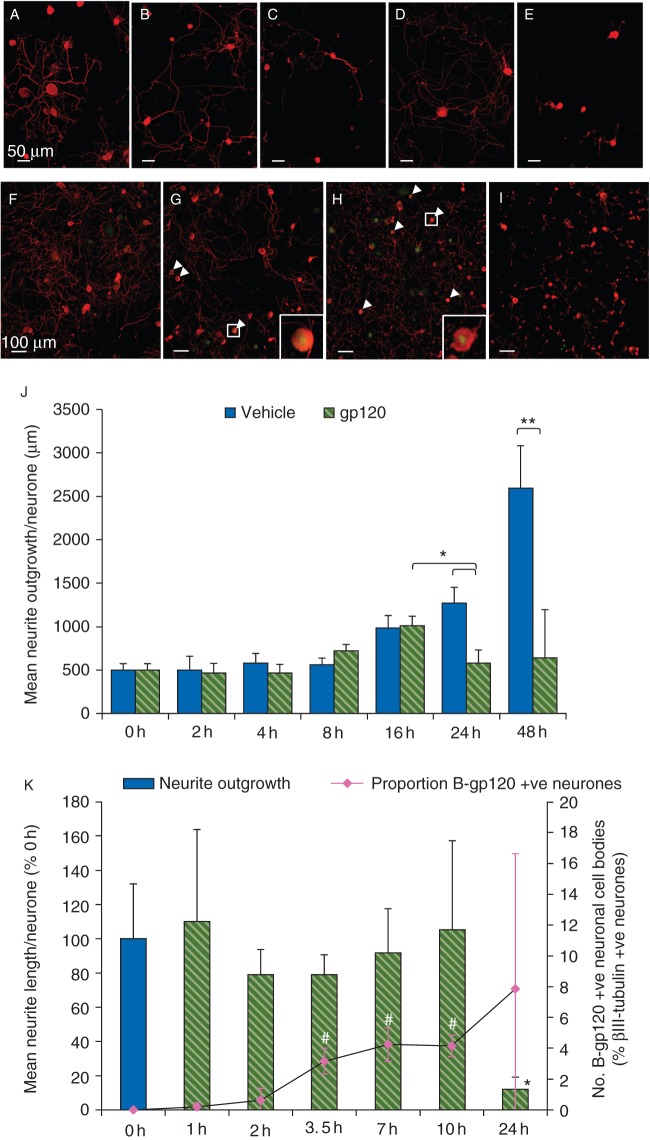
Temporal course of gp120-associated neurite degeneration and accumulation of biotinylated-gp120_MN_ in the neuronal cell body. Untreated cultures were fixed at the time of application (*t*=0 h) to assess baseline neurite outgrowth. Remaining cultures were treated with vehicle or 2 nM gp120_MN_ or, in a separate experiment, 2 nM biotinylated-gp120_MN_ (B-gp120_MN_) in C-DMEM. (a–i) Representative images of untreated DRG cultures at *t*=0 h (a and f) and treated DRG cultures after 24 h (b and c) or 48 h (d and e) exposure to vehicle (b and d) or 2 nM gp120_MN_ (c and e), or those treated with B-gp120_MN_ after 3.5 (g), 10 (h), or 24 h (i). Treated cultures were fixed at set time-points after application, and immunostained for βIII-tubulin (red) to complete neurite analysis. (j) Quantification of the temporal course of neurite outgrowth, and (k) its association with the temporal localization of B-gp120_MN_ with neuronal cell bodies. White arrowheads indicate neuronal cell bodies positive for B-gp120_MN_ immunolabelling (green). Inset shows ×5.2 magnification of indicated white box. Data presented as mean and (sd). *P<0.05 and **P<0.01 *vs* 0 h baseline, and ^#^*P*<0.05 vs the respective baselines using two-factor ANOVA; *n*=6. Percentage (%) of baseline was used to normalize across the two biological replicates. Scale bars: (a–e) 50 µm and (f–i) 100 µm.

Neurite outgrowth in gp120_MN_- and vehicle-treated adult DRG cultures were similar for the first 16 h after treatment (Fig. 1j). After 24 h, neurite outgrowth in gp120-treated cultures was significantly less than both vehicle-treated cultures after 24 h [mean neurite outgrowth per neurone (NOPN) (sd), gp120 24 h: 580 (260) µm *vs* vehicle 24 h: 1270 (308) µm; *P*<0.05; Fig. 1b, c, and j], and gp120-treated cultures at the earlier 16 h time point [gp120 16 h: 102 (179) µm; *P*<0.05]. This was maintained until the study end [gp120 48 h: 643 (950) µm *vs* vehicle 48 h: 2600 (690) µm; *P*<0.05; Fig. 1d, e and j).

The temporal course of neurite degeneration was studied in parallel to biotinylated-gp120_MN_ (B-gp120_MN_) (Fig. 1f–i and k). B-gp120_MN_ induced a similar profile of neurite degeneration to the unbiotinylated form, confirming that biotinylation does not interfere with the biological activity of gp120. B-gp120_MN_ was detectable in a small proportion of neurones within 1 h of application [0.2 (0.3)%; Fig. 1g], before neurite degeneration became evident. Neuronal accumulation of B-gp120_MN_ peaked at 24 h [7.9 (8.7)%; Fig. 1k], when significant neurite degeneration first became evident.

### gp120-exposed BMDM-released mediators reduce neurite outgrowth

**Fig 2:**
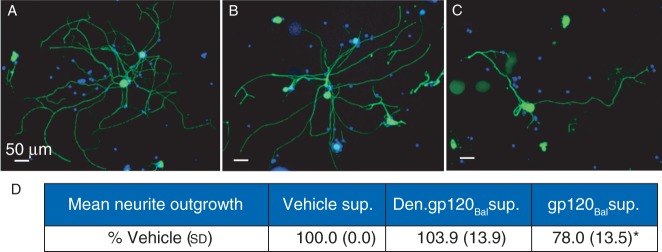
Neurite analysis after exposure to conditioned BMDM media. (a–c) Representative images of primary DRG cultures immunolabelled for βIII-tubulin (green) and nuclei counterstained with Hoechst 32258 (blue). One-day-old DRG cultures were treated for 24 h with DMEM/F-12 supplemented 1:1 with culture media from BMDMs stimulated for 4 h prior with (a) vehicle, (b) 2 nM denatured-gp120_Bal_, or (c) 2 nM gp120_Bal_. Scale bars=50 µm. DRG cultures treated with culture media from gp120_Bal_-treated BMDMs showed a significant reduction in the mean NOPN, as a percentage of vehicle-treated cultures (d). Conditioned media from denatured-gp120_Bal_-treated BMDMs induced no change in neurite outgrowth relative to vehicle control. Data presented as mean (sd). *P<0.05 *vs* vehicle-treated cultures, using one-way anova and Tukey's *post hoc* analysis (*n*=5–6; five to six technical replicates across two biological studies).

Adult DRG cultures treated for 24 h with supernatant from gp120_Bal_-conditioned BMDMs showed significantly less neurite outgrowth relative to those treated with vehicle-conditioned supernatant [mean NOPN relative to vehicle (100.0%) (sd), gp120-BMDM: 78 (14)%, *P*<0.01; Fig. 2a–d]. In contrast, denatured-gp120_Bal_ BMDM media had no significant effect on neurite outgrowth [den.gp120-BMDM: 104 (14)%, *P*=0.448; Fig. 2a, b, and d]. Furthermore, neither additional negative control (cell-free, 24 h gp120-treated DMEM media, and naïve, cell-free, gp120-free DMEM media) caused any changes compared with vehicle control (data not shown). This confirms that neither the culture condition nor non-specific effects of gp120 were responsible for the responses seen.

**Fig 3:**
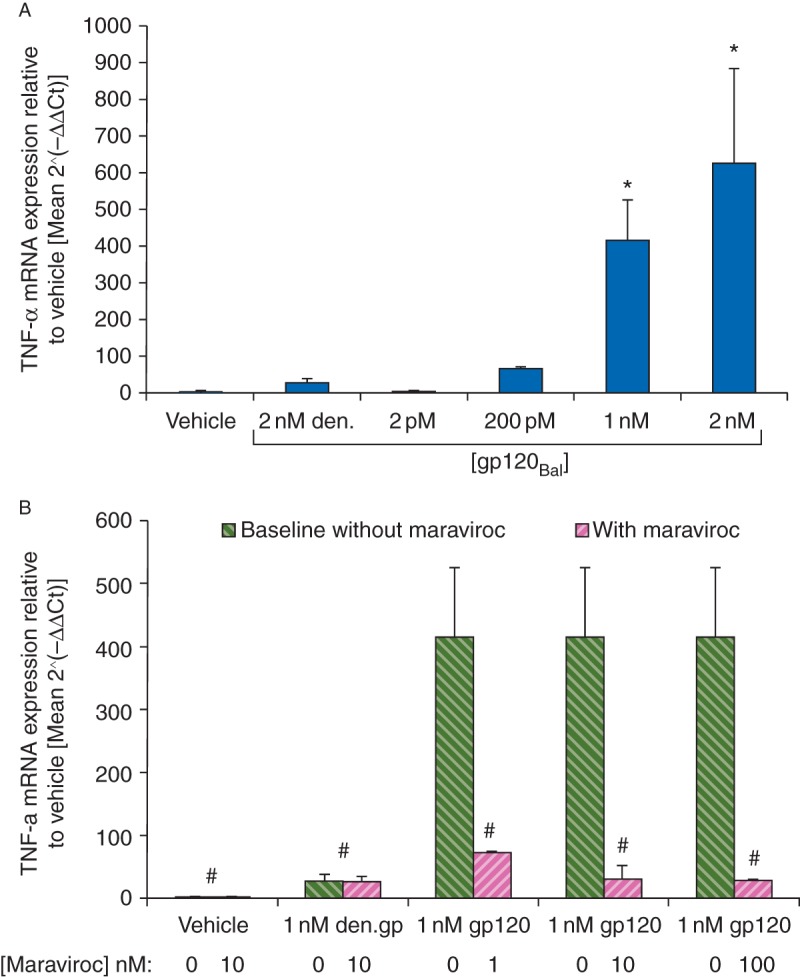
TNF-α expression changes in BMDMs after gp120 exposure and inhibition with the CCR5 antagonist maraviroc. (a) gp120_Bal_ induced a concentration-dependent increase in TNF-α expression after 4 h stimulation of BMDMs with vehicle, 2 nM denatured-gp120_Bal_ or 2, 200 pM, 1 or 2 nM gp120_Bal_, in IMDM. Data presented as mean 2^−^^ΔΔCt^-fold change (sd), *n*=4. *P<0.05 *vs* vehicle- and den.gp120-treated control levels using one-way anova and Tukey's *post hoc* analysis. (b) Primary BMDM cells were pretreated for 1 h with either vehicle control or 10 nM maraviroc and then exposed for 4 h to either vehicle, 1 nM denatured-gp120_Bal_ ,or 1 nM gp120_Bal_ diluted in IMDM or presence of 1, 10, or 100 nM maraviroc. Changes in TNF-α mRNA gene expression were assessed relative to vehicle control. Data presented as mean 2^−^^ΔΔCt^-fold change (sd), *n*=4. *P<0.05 *vs* 1 nM gp120-treated baseline values using two-way anova and Tukey's *post hoc* analysis.

After our evidence for gp120-induced, macrophage-mediated neurotoxicity, we assessed TNF-α mRNA expression in BMDMs using qPCR to clarify macrophage responses to gp120. gp120_Bal_ induced TNF-α mRNA expression in BMDMs in a concentration-dependent manner that was significant at concentrations of 0.2, 1, and 2 nM (65-, 415-, and 628-fold increase, respectively) relative to vehicle- and denatured-gp120_Bal_-treated cultures (1- and 27-fold increase, respectively; *P*<0.05; Fig. 3a). No significant increase in TNF-α was noted in BMDMs treated with denatured-gp120_Bal_ [den.gp120 2 nM: 27 (11) fold, *P*>0.05].

Using the highly selective CCR5 antagonist maraviroc, we confirmed that gene expression changes were initiated via specific, biologically active binding of gp120_Bal_ to the CCR5 receptor. Preincubation and continued presence of 1 nM maraviroc was sufficient to significantly attenuate gp120-induced TNF-α expression (Fig. 3b). Application of 10 and 100 nM maraviroc reduced TNF-α expression levels further, but neither fully ablated the response. The plateau of inhibition reached was on par with that seen with denatured-gp120_Bal_. Application of 10 nM maraviroc alone showed no induction or suppression of TNF-α expression.

### gp120 up-regulates a pro-inflammatory gene expression profile in BMDMs

gp120_Bal_ significantly induced the expression of 25 genes in BMDM cultures 4 h after application (Table 1), CXCL11, interleukin (IL)-27, inducible nitric oxide synthase (iNOS), IL-1α, IL-1β, TNF-α, CCL2, and prostaglandin (PG) E synthase (3200-, 2400-, 2200-, 2000-, 950-, 310-, 130-, and 47-fold change relative to vehicle, respectively). A further 12 genes showed increased expression but were not statistically significant (Supplementary Table S2). A further eight genes showed decreased expression that did not reach statistical significance (Supplementary Table S2). In contrast, no expression changes with denatured-gp120-treated BMDM samples were statistically significant.

**Table 1:** Relative gene expression of selected cytokines. Mean 2^−ΔΔCt^-fold change (2^−ΔΔCt^ standard range) of all gene expression profiles affected by gp120_Bal_ exposure (*n*=4; two biological repeats with two technical replicates in each). Data from BMDMs treated with 2 nM denatured-gp120_Bal_ or 2 nM gp120_Bal_ in IMDM compared with the vehicle control. Values were normalized to the mean of four housekeeping genes. *P<0.05; **P<0.01; ***P<0.001; all refer to gp120-treated fold changes only. No fold changes for denatured-gp120_Bal_ were statistically significant. Δ denotes cases where expression became detectable in more than one-fourth, but <4/4, samples and compared with samples from vehicle- and denatured-gp120_Bal_-treated BMDMs where expression was generally undetectable.

Target	Fold difference in den.gp120 relative to vehicle	Fold difference in gp120 relative to vehicle	*P*-value
Up-regulated			
CXCL11	5.9 (1.6–22.5)	3205.5 (2241.2–4583.4)	***
IL-27	6.5 (3.8–11.3)	2446.1 (1857.3–3221.5)	*
iNOS	2.0 (0.7–5.7)	2244.0 (1846–2728)	***
CXCL9	2.2 (0.9–5.2)	2082.9 (1525–2844)	***
CXCL2	10.0 (3.2–31.4)	2060.5 (1186.4–3578.6)	*
IL-1α	8.9 (3.2–25.2)	2016.3 (1336–3043)	***
CCL12	12.4 (0.4–374.5)	1167.2 (1005–1355)	*
IL-1β	8.6 (5.4–13.6)	948.3 (798.4–1126)	***
CXCL10	5.1 (2.2–11.4)	939.9 (737.5–1198)	***
CCL5/RANTES	0.8 (0.2–3.1)	474.5 (342.2–657.9)	**
TNF-α	2.4 (0.7–8.6)	313.7 (199.1–494.3)	***
CCL4	2.5 (1.0–6.2)	306.3 (205.7–456.1)	**
CCL20	1.7 (0.7–3.8)	276.6 (133.9–571.6)	***
CXCL1	3.9 (2.2–6.9)	248.0 (198.5–309.9)	***
CCL2	2.1 (1.0–4.4)	134.8 (107.9–168.4)	***
CCL3	1.4 (0.2–9.0)	127.1 (53.9–299.9)	**
CCL7	1.4 (0.8–2.5)	93.4 (81.8–106.6)	***
COX2	0.3 (0.1–0.9)	57.2 (43.1–76.0)	
PGES	1.1 (0.2–6.4)	46.8 (15.4–142.2)	*
IL-15	1.4 (0.7–2.8)	43.1 (30.1–61.7)	**
CX3CL1	1.6 (0.9–2.8)	41.4 (22.8–75.1)	***
LIF	1.4 (0.3–5.9)	23.0 (21.6–24.5)	
CXCL6	2.0 (0.8–4.7)	20.6 (5.3–80.9)	*
IL-6	1.3 (0.07–23.5)	18.6 (1.3–266.0)	
CCL19	0.8 (0.6–1.1)	14.0 (8.7–22.5)	**
CCL9	1.08 (0.4–2.2)	7.5 (4.3–13.0)	*
Ereg	1.3 (0.5–3.3)	6.5 (3.6–11.7)	
IL-12a	No change	4.6 (1.1–19.5)	
CXCL13	0.9 (0.5–1.7)	3.2 (1.2–8.2)	
Edn1	0.9 (0.5–1.5)	3.1 (2.0–4.9)	
M-CSF1	1.2 (0.9–1.6)	2.9 (1.9–4.4)	*
IL-18	1.3 (0.6–2.6)	2.9 (1.9–4.3)	
CXCL16	1.1 (0.7–1.7)	2.3 (1.8–2.9)	*
IL-12β	No change (0/4 detected)	Increase [2/4 *µ*=33.6 (0.1)]	Δ
Csf2	No change (0/4 detected)	Increase [2/4 *µ*=33.8 (0.1)]	Δ
IL-23α	No change (0/4 detected)	Increase [2/4 *µ*=33.8 (0.5)]	Δ
CCL17	Increase (1/4 34.91)	Increase [2/4 *µ*=34.7 (0.6)]	Δ
Down-regulated			
Kitlg	0.8 (0.2–2.9)	0.2 (0.1–0.5)	
Artn	0.3 (0.2–0.4)	0.3 (0.2–0.4)	

### Effect of TNF-α on DRG neurite outgrowth

**Fig 4:**
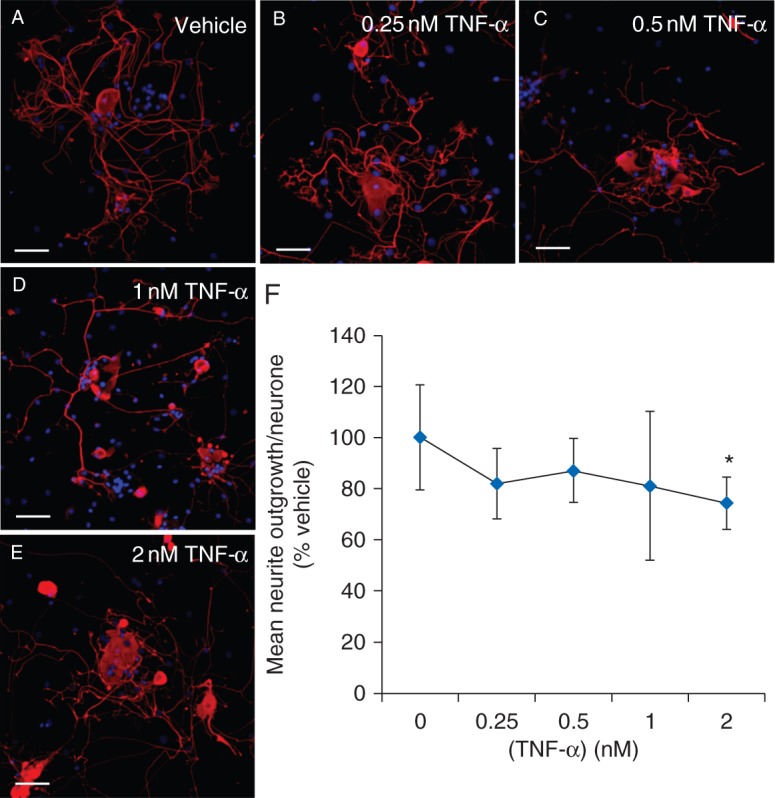
Effect of TNF-α on neurite outgrowth in primary adult DRG cultures. Representative images of adult primary DRG cultures treated for 24 h with vehicle (a) or 0.25 nM (b), 0.5 nM (c), 1 nM (d), and 2 nM (e) TNF-α in C-DMEM. Addition of TNF-α induced reduced neurite outgrowth that was significant at 2 nM (f). Data presented as mean neurite outgrowth/neurone (sd), after 24 h treatment, normalized to (percentage of) the mean neurite outgrowth/neurone of vehicle-treated cultures (*n*=3–9). *P<0.05 using one-way anova and Tukey's *post hoc* analysis. Scale bars=50 µm.

DRG cultures were exposed for 24 h to vehicle or TNF-α (0.25–2 nM), using concentrations based on the CSF and plasma levels found in HIV patients exhibiting SN.^16^ TNF-α induced a reduction in neurite outgrowth relative to vehicle that decreased with increasing concentration [0.25 nM: 82% (14), 0.5 nM: 87% (12), 1 nM: 81% (29) of vehicle; *P*>0.05; Fig. 4] and reached significance at 2 nM [74% (10) of vehicle; *P*<0.05]. We also assessed cell survivability by quantifying the proportion of neurones that exhibited signs of apoptosis, by counting condensed and fragmented Hoechst-labelled nuclei. No significant difference was observed between vehicle-treated cultures [45 (3.2)% neurones] and those exposed to 2 nM TNF-α [48 (0.9)% neurones].

## Discussion

We showed that direct gp120 neurotoxicity to sensory neurones does not appear to be a predominant mechanism underlying HIV-SN, but rather macrophages are probably important in HIV-SN pathogenesis. Thus, treatment with supernatant from gp120-exposed BMDMs of DRG neurones reduced neurite outgrowth. We also found 25 cytokine and chemokine genes up-regulated in gp120-exposed BMDMs, some of which are already known for their roles in neuropathic pain,^16^ further supporting the possible involvement of macrophages in HIV-SN pathogenesis. Finally, application of the clinically available CCR5 antagonist maraviroc attenuated TNF-α production by gp120-exposed BMDMs, highlighting its potential for preventing HIV-SN.

### gp120–neuronal interaction is unlikely the direct cause of neurite outgrowth reduction

Our experiments present for the first time the temporal profile of gp120-associated neurotoxicity. The temporal course of B-gp120_MN_ binding showed an interaction with DRG neurones within 1 h of exposure. However, the maximum proportion of DRG neurones with evidence of B-gp120_MN_ accumulation was about 8% after 24 h, despite reduced neurite outgrowth in the global population of neurones. We used 2 nM gp120 in our experiments, a concentration in the higher range of that used by others.^7,8,17^ However, in the light of a reported *K*_d_ ranging from 4 to 300 nM for gp120 binding to CXCR4 and CCR5 in the absence of CD4,^18–20^ it is more likely that the majority of these effects were mediated via intermediary cells, such as macrophages, which were also present in our cultures (Supplementary Fig. S3). Studies on axonal degeneration are starting to draw insight from mechanisms underlying Wallerian degeneration.^21^ After the introduction of the slow Wallerian degeneration mouse mutant, it is now believed that neurite degeneration is not a passive process but instead an active process that becomes activated once a threshold of parameters is met. We believe that direct gp120 neurotoxicity is unlikely to fully explain the global extent of neurite toxicity seen in our studies.

### Macrophage-released mediators reduce neurite outgrowth

Many studies have explored the involvement of Schwann cells in gp120-mediated neurotoxicity^8,17^ and of microglia in HIV-associated dementia,^22^ but the effects of gp120 on peripheral macrophages, and subsequent association with neurite degeneration, have been less well covered. We found that supernatant from BMDMs treated with gp120_Bal_, a CCR5-macrophage-selective gp120 strain, induced significant neurite toxicity suggesting neurotoxic factors were secreted by BMDMs as a result of gp120_Bal_ exposure. With the indication that macrophage-released mediators were neurotoxic to DRG cultures, we performed qPCR analysis to assess changes in TNF-α mRNA expression. Given the induced transcription of TNF-α, along with other known algogenic mediators (IL-1β, CCL5) in gp120-exposed BMDMs, we propose these among the factors mediating the neurotoxicity observed. Although we did not confirm mediator release at a protein level, release of TNF-α, IL-1β, and CCL5 has been previously demonstrated in gp120-stimulated macrophage cultures.^23^ Our data are consistent with human data showing infiltrated macrophages and concomitant presence of pro-inflammatory cytokines in the DRG of AIDS patients with a history of HIV-SN,^10^ and the extent of axonal degeneration correlated with the level of macrophage infiltration.^24^

Maraviroc demonstrates potent antiviral activity against all CCR5-tropic HIV-1 viruses tested (geometric mean 90% inhibitory concentration of 2 nM); its mechanism of action has been established using cell-based assays, where it blocks binding of gp120 to CCR5 to prevent the membrane fusion events necessary for viral entry.^25^ This has led to its use as an effective antiretroviral treatment.^26^ Even at the lowest concentration tested (1 nM), maraviroc almost completely ablated the gp120-induced gene expression of TNF-α. Similar effects have been demonstrated in cultured microglia, cells that have a similar lineage to macrophages.^22^ This confirms that M-tropic gp120_Bal_ induces mRNA changes via selective activation of CCR5. The expression changes were not completely ablated, even with 100 nM maraviroc. However, the apparent plateau reached matched the expression changes seen with denatured-gp120_Bal_. Given that maraviroc alone, in the absence of gp120, had no effect on BMDM TNF-α expression, we propose that these low level changes are evidence of a non-specific, CCR5-independent, antigenic macrophage response to the denatured, but still highly glycosylated foreign glycoprotein.^27^ It has not yet been investigated whether maraviroc protects against gp120-induced neurite degeneration or associated pain, which was unfortunately outside the scope of our studies.

### Cytokine/chemokine up-regulation in gp120-exposed macrophages

Our study is the first to assess changes to a selection of genes known for their roles in inflammation, immune responses, and HIV-SN in peripheral macrophages. Among the 95 genes assessed, those extensively induced by gp120_Bal_ were CXCL11, CXCL9, and CXCL10, IL-27, and iNOS, a profile typical of an M1 pro-inflammatory macrophage response,^28^ and also IL-1α, IL-1β, TNF-α, CCL2, and PG E synthase, which have all been shown to play a key role in neurodegeneration and pain.^29–31^ Our data suggest the release of end products of these genes when up-regulated in gp120-exposed BMDMs, which supports reports of M1 phenotypic states being neurotoxic to cortical neuronal cultures, while M2 are neuroprotective.^32^ Some key functions of up-regulated genes are: (i) IL-18, 21, 27, and 32 cytokines and CXCL9–11 and CCL2 chemokines have potent effects on lymphocyte trafficking allowing further immune cell recruitment;^30,33^ (ii) IL-1α and 1β cytokines are associated with neurotoxicity via microglia and macrophages^34^ and with neuronal sensitization and hyperalgesia;^35^ (iii) iNOS produces nitric oxide that can cause deleterious effects on neighbouring cells; (iv) COX-2 is a macrophage-derived enzyme and together with PGE is implicated in many neurodegenerative diseases and HIV infection.^36,37^

In summary, we have shown the key involvement of macrophages in gp120-induced neurotoxicity *in vitro*. It is likely that the interaction between macrophages and the peripheral nervous system, which could happen at an early stage of HIV infection when the viral load is high, the immune response is robust, and ART is not yet initiated, is important in the development of HIV-SN. Our study presents a plausible therapeutic strategy for gp120-induced neurotoxicity by blocking the upstream interaction of gp120 with macrophages expressing CCR5. Future experiments will explore the therapeutic potential of maraviroc *in vitro* to prevent axonal degeneration and *in vivo* to prevent neuropathy in rodent models and in SIV-infected non-human primates, and also in clinical studies to follow up patients treated with maraviroc and see if they develop symptoms and signs of neuropathy.

## Supplementary material

Supplementary material is available at *British Journal of Anaesthesia* online.

## Authors' contributions

P.J.M.: performed the study and reviewed the manuscript; W.H.: partly involved in the design of the study, reviewed the study results, and wrote the manuscript; J.D.: assistance running the qPCR array and reviewing the manuscript; K.O. and S.B.M: study design and review of the manuscript; A.S.C.R: conceived the study, oversaw design, conduct and analysis, and contributed to writing. All authors participated in revision of the manuscript.

## Declaration of interest

None declared.

## Funding

P.J.M. was a recipient of a PhD studentship from the London Pain Consortium, which is funded by a Wellcome Trust Strategic Award (ref. 083259). A.S.C.R., S.B.M., and W.H. are also funded by the Innovative Medicines Initiative Joint Undertaking, under grant agreement no. 115007, resources of which are composed of financial contributions from the European Union's Seventh Framework Program (FP7/2007-2013) and EFPIA companies' in kind contributions.

## Supplementary Material

Supplementary Data
