# Supplementary material for: Macrophage–sensory neuronal interaction in HIV-1 gp120-induced neurotoxicity
Source: Br J Anaesth. 2014 Sep 16;114(3):499–508. doi: 10.1093/bja/aeu311 (PMC4332570; doi:10.1093/bja/aeu311)
Supplement: Supplementary Data [file supp_114_3_499__index.html]

Macrophage–sensory neuronal interaction in HIV-1 gp120-induced neurotoxicity — Macrophage–sensory neuronal interaction in HIV-1 gp120-induced neurotoxicity — Macrophage–sensory neuronal interaction in HIV-1 gp120-induced neurotoxicity — Supplementary Data 

# Macrophage–sensory neuronal interaction in HIV-1 gp120-induced neurotoxicity

## Supplementary Data

Supplementary Data

**Files in this Data Supplement:**

- Supplementary Data - Doc file
