# Supplementary material for: Macrophage–sensory neuronal interaction in HIV-1 gp120-induced neurotoxicity
Source: Br J Anaesth. 2014 Sep 16;114(3):499–508. doi: 10.1093/bja/aeu311 (PMC4332570; doi:10.1093/bja/aeu311)
Supplement: Supplementary Data [file supp_aeu311_aeu311supp.doc]

Macrophage-sensory neuronal interaction in HIV-1 gp120-induced neurotoxicity

Supplementary Materials

**1. Additional methods**

**1.1) Reagent details**

The following compounds were used in the study: gp120_MN_ (ImmunoDiagnostics, USA), biotinylated gp120_MN_ (ImmunoDiagnostics, USA), gp120_Bal_ (a gift from Prof Maria Papathanasopoulos, University of the Witwatersrand, Johannesburg, South Africa), tumour necrosis factor alpha (TNF-α; GIBCO, UK), Maraviroc (NIH AIDS Reagent Program, USA), interleukin 4 (IL-4; Peprotech, UK), eicosapentaenoic acid (EPA; Sigma, UK). Gp120 was denatured by incubating stock vials of gp120_MN_ or gp120_Bal_ (10µl at 100ng µl^-1^ in 0.1% BSA [Sigma, UK]/PBS) for 1hr at 90ºC, just prior to treatment preparation and application, to serve as a control. Vehicle treatment always used the same solvent composition used to dilute and prepare the denatured-gp120 and gp120 treatments within a study, i.e. 0.1% bovine serum albumin/phosphate buffered saline.

1.2) Choice of gp120 protein

Gp120_MN_ is a dual-tropic form that targets both CXCR4 and CCR5 receptors, both of which are expressed by DRG neurons. Therefore, this form was used for our neuronal cultures to assess gp120-associated neurotoxicity. However, HIV strains preferentially target macrophages via their CCR5 receptors. Therefore gp120_MN_, which is thought to show slight preference for CXCR4, is not appropriate for these experiments and gp120_Bal_, an M-tropic CCR5-selective form, was used instead for experiments assessing macrophage responses to gp120.

**1.3) Culture media**

For DRG cultures, completed-Dulbecco's Modified Eagle Medium comprised C-DMEM (DMEM [Invitrogen, UK], 10% foetal bovine serum [FBS; GIBCO, UK] and supplemented with NGF [50ng ml^-1^; Sigma, UK] and aphidicolin [10µM; Sigma, UK]. Poly-L-lysine- (0.1mg ml^-1^; Sigma, UK) and laminin (10µg ml^-1^; Invitrogen, UK) were used to coat coverslips. For macrophage cultures, macrophage complete medium comprised C-IMDM (Iscove’s Modified Dulbecco’s Medium [Invitrogen, UK] with 10% FBS) supplemented with macrophage colony-stimulating factor (M-CSF, 5ng ml^-1^; ProSpec, UK). All culture media contained penicillin/streptomycin antibiotics [1:100, Sigma, UK].

**1.4) Immunocytochemistry in primary DRG cultures**

Cells were permeabilised with 0.1% Triton X-100 then incubated for 1.5h with a relevant primary antibody: mouse anti-βIII-Tubulin (1:2500; Promega, UK) for neurones, rabbit anti-GAP-43 (1:1000; Chemicon, UK) for neurones, and mouse anti-rat ED1 (CD68) (1:500; Millipore, UK) for macrophages. Following incubation with antisera, cultures were washed with PBS then incubated for 1h at room temperature, with a relevant fluorescently-labelled secondary antibody or streptavidin compound: donkey anti-mouse Cy3 (1:1000; Jackson ImmunoResearch, USA), anti-mouse FITC (1:500; Jackson ImmunoResearch, USA), FITC-streptavidin (3.6μg ml^-1^; Jackson ImmunoResearch, USA), goat anti-rabbit Alexa Fluor 488 (1:400; Invitrogen, UK), and goat anti-mouse Alexa Fluor 568 (1:400; Invitrogen, UK). Both primary and secondary antibodies were diluted in 10% donkey serum/PBS. Nuclei were counter-stained with Hoechst 32258 (1:10,000; Sigma, UK). Cells were mounted onto glass slides and analysed on a Nikon 80i microscope. Images were acquired, under blinded conditions, with a digital Nikon DXM1200F camera-controlled with LUCIA G software (Laboratory Imaging, CZ). Neurite analysis was performed using CSIRO HCA-vision software ([www.hca-vision.com](http://www.hca-vision.com/)). The experimenter was unblinded to treatment only after data had been analysed.

**1.5) DRG culture treatment with conditioned macrophage media or TNF-α**

Macrophage media was collected 4h after vehicle, denatured-gp120 or gp120 treatment and spun through a 50kDa Vivaspin column at 1500rpm to remove cells and compounds larger than 50kDa including excess gp120. Cell-free supernatant was collected and diluted 1:1 with 2x concentrate DRG media (C-DMEM supplemented with NGF and aphidicolin) to make it ready for use on cultured neurones. Media from 1-day old DRG cultures was removed and replaced with 500µl of (vehicle-, denatured-gp120, gp120_Bal_) macrophage-conditioned media and incubated at 37°C. After 24h, DRG cultures were fixed and immunostained.
In a separate experiment, 1 DIV DRG cultures were treated with vehicle or TNF-α (0.25, 0.5, 1, or 2nM in DMEM) for 24h, and then processed for immunocytochemistry. Cell survivability was assessed by quantifying the proportion of neurones that exhibited signs of apoptosis, namely condensed and fragmented Hoechst-labelled nuclei.

1.6) DRG neurite outgrowth analysis

Following immunostaining, samples within the same treatment were mounted in triplicate on the same microscope slide and a blinding procedure was implemented. Treatment labels on each slide were covered with a blank label, and then randomly re-labelled with A, B, C and so on, by someone different to the experimenter. The images were acquired by the experimenter from each slide in alphabetical order. To prevent bias in image acquisition, selection criteria were established. Between five and ten images were taken at specific points on each coverslip, to get an unbiased representation and allow standardisation of acquisition across all samples (Supplementary Fig. 1). Images were acquired at 10x magnification with a screen size of 1180x920µm or 1128.7x957.9pixels. This relates to a conversion of 0.95877µm per pixel. CSIRO HCA-vision software was used for all neurite outgrowth analysis ([www.hca-vision.com](http://www.hca-vision.com/)). HCA-vision has been previously validated for neurite analysis.[^1^](#_ENREF_1) The software requires the experimenter to set the parameters within three main categories, Neurone Body Detection, Neurite Detection and Neurite Analysis. The settings were kept constant within studies. Manual checks were also performed to validate the accuracy of the HCA analysis obtained. We obtained detail information on neuronal outgrowth, including neurone numbers, total and maximum neurite length, total, maximum and/or mean branch points, roots, segments, extremities and intensity of immunostaining. However, for the purposes of our experiment we used ‘average neurite outgrowth per neurone’ as our endpoint. Average neurite outgrowth per neurone was calculated per coverslip by taking the sum of the total neurite lengths detected in each image and dividing by the total number of neuronal cell bodies identified (Supplementary Fig. 2). This was done for 5–10 images per coverslip to get an average neurite length per neurone for each technical replicate (each coverslip within the same treatment group). Each experiment (biological replicate) took the average across three technical replicates. This was repeated to obtain data from 2–3 biological replicates. The average value calculated from the biological replicates formed the final ‘mean neurite outgrowth per neurone’ values presented in this manuscript. This method assumes that gp120 affects globally on all neurones and avoids the wide and potential inaccurate variation that can occur if the total neurite length of each neurone as identified by HCA-vision was used, without normalisation. However, this method does risk masking select effects on neuronal subtypes. Experimenter remained blinded to treatment throughout image acquisition and neurite analysis and was only unblinded at the end in order to do statistical and post-hoc analysis.

Supplementary Fig. 1

Supplementary Fig. 1. Example coverslip and markers indicating standardised positions for image acquisition.

Supplementary Fig. 2


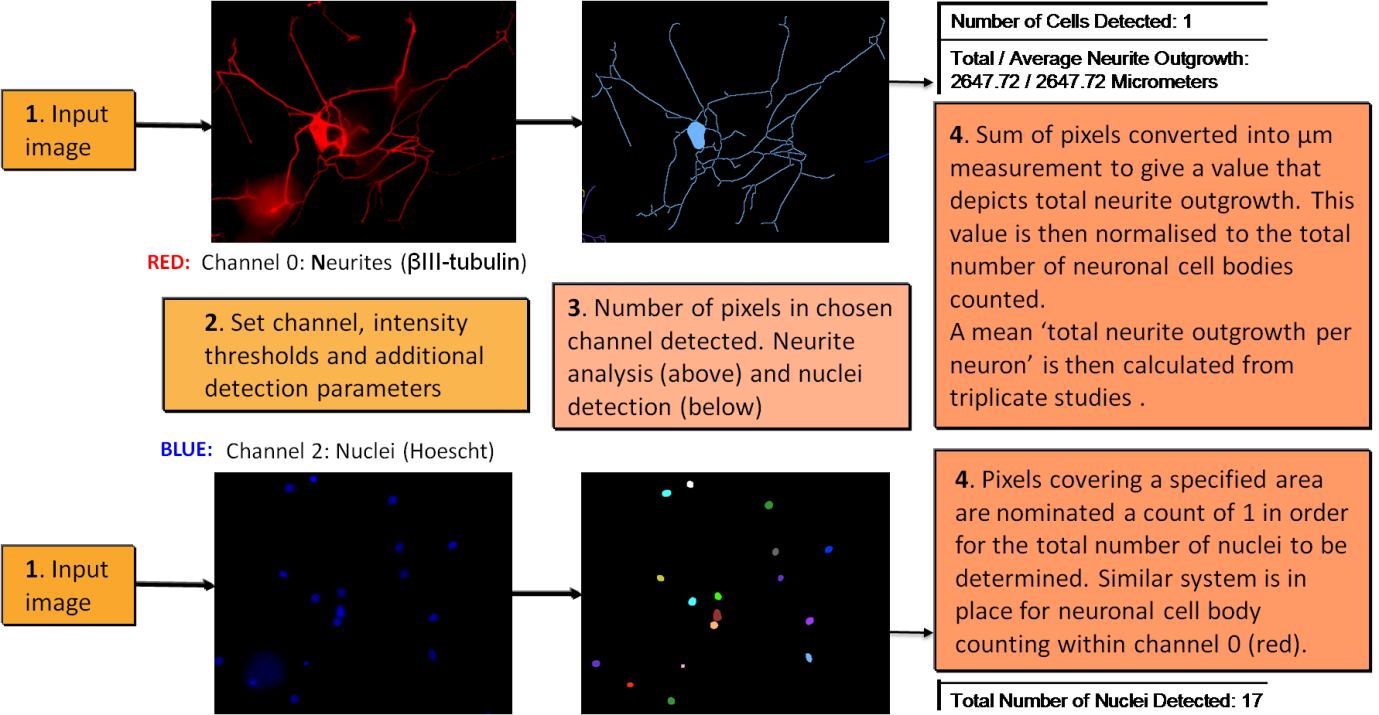


**Supplementary Fig. 2. Flow chart depicting steps for neurite analysis using HCA vision analysis software.**

**1.7) cDNA synthesis, reverse transcription, and quantitative real-time polymerase chain reaction (qPCR)**

All procedures were performed according to manufacturer’s instructions. Total RNA was extracted using the peqGOLD MicroSpin Total RNA Kit (PeqLab, UK). Reverse transcription was performed using the First-Strand SuperScript® III First-Strand System (Invitrogen, UK). All PCR reactions were performed in duplicate or triplicate per sample using with Fast SYBR green master mix (Applied Biosystems, UK) in 20µl reactions. Reactions were processed on a 96-well StepOnePlus™ Real-Time PCR instrument, using the comparative Ct method, normalised to the endogenous housekeeping genes GAPDH (glyceraldehyde-3-phosphate dehydrogenase) or YWHAZ (14-3-3 protein zeta/delta) and assessed relative to the calibrator (untreated or vehicle-treated cultures). Cts>38 were considered to be false amplification readings. Primer sequences are in Supplementary Table 1. Melt curves were performed immediately following qPCR cycling to verify primer specificity for its target gene.

Supplementary Table 1. Characteristics of primers for selected targets and housekeeping genes.

| Gene Symbol | Forward Primer | Reverse Primer | Reaction Conc. (μm) | Amplicon Size (bp) |
| --- | --- | --- | --- | --- |
| TNF-α | ATCCGAGATGTGGAACTGGCA | CCGGACTCCGTGATGTCTAAGT | 0.2 | 659[^2^](#_ENREF_2) |
| CCL2 | ATGCAGTTAATGCCCCACTC | ATGCAGTTAATGCCCCACTC | 0.5 | 167[^3^](#_ENREF_3) |
| iNOS | CACCTTGGAGTTCACCCAGT | ACCACTCGTATTGGGATGC | 0.5 | 167[^4^](#_ENREF_4) |
| Arg-1 | TATCGGAGCGCCTTTCTCTA | ACAGACCGTGGGTTCTTCAC | 0.5 | 189[^4^](#_ENREF_4) |
| IL-6 | TCCTACCCCAACTTCCAATGCTC | TGGATGGTCTTGGTCCTTAGCC | 0.4 | 79[^5^](#_ENREF_5) |
| IL-1β | CACCTCTCAAGCAGAGCACAG | GGGTTCCATGGTGAAGTCAAC | 0.4 | 79[^5^](#_ENREF_5) |
| GAPDH | TGCACCACCAACTGCTTAGC | GGCATGGACTGTGGTCATGAG | 0.5 | 87 |
| YWHAZ | GCTACTTGGCTGAGGTTGCT | TGCTGTGACTGGTCCACAAT | 0.5 | 61[^6^](#_ENREF_6) |

**1.8) Intradermal injection of gp120**

Ipsilateral plantar paws were intradermally injected with 20µl sterile PBS containing either 400ng denatured-gp120_Bal_ (Sham) or 400g gp120_Bal_. Tissue was freshly harvested 5hr following intradermal injection. The expressions of five key cytokines (iNOS, TNF-α, CCL2, IL-1β, and IL-6) were examined. We chose iNOS is because the induction of enhanced iNOS expression is suggestive of macrophage infiltration due to the predominantly macrophage-specific expression of iNOS. We chose TNF-α, CCL2, IL-1β, and IL-6 is because previous *in vivo* rat studies have revealed increased expression of these cytokines and chemokines following intrathecal injection of and perineural treatment with gp120, which was associated with neuropathic pain-like behaviours.

**1.9) Immunoregulation of macrophage response**

BMDMs were primed with IL-4 for 24h prior to treatment experimentation. In brief, after 8 DIV, primary BMDM culture media was replaced with serum-free IMDM, in the absence or presence of 5ng ml^-1^ IL-4. Media was replaced 24h later with vehicle, 1nM denatured-gp120_Bal_ or 1nM gp120_Bal_ in 1ml IMDM. For EPA studies, vehicle or 2nM gp120_Bal_ was added in the absence or presence of 0.08, 0.8 or 8 EPA in 1ml IMDM, to serum-deprived primary BMDMs at 9 DIV. 1nM denatured-gp120_Bal_ without EPA was an additional control treatment. For both studies, RNA was extracted 4hr after treatment, as outlined previously, and mRNA expression assessed relative to either unprimed, vehicle-treated BMDMs or vehicle-treated BMDMs in absence of EPA, respectively. M1 and M2 phenotypes were confirmed by increases in iNOS and Arg1 mRNA expression, respectively. The iNOS:Arg1 ratio was also used, presented as a percentage of the vehicle iNOS:Arg1 expression ratio. A reduction in iNOS:Arg1 relative to vehicle indicated a significant increase in iNOS expression (M1 phenotype), while a higher ratio relative to vehicle indicated a significant increase in Arg1 expression (M2 phenotype).

**2. Additional results**

### 2.1) Cytokine microarray

The effect of denatured-gp120 and gp120 on the expression of 92 genes analysed in the custom-made qPCR array, relative to vehicle, are shown in Supplementary Table 2. Where expression was undetermined in 1 of the 4 sample replicates, the mean Ct value was calculated from the remaining 3 data values and undetected samples were excluded. Where expression was undetermined in more than 1 but less than 4 of the 4 sample replicates, the undetermined sample was given a default Ct of 38, unless this occurred in both vehicle-control or treatment-treated, in which case no fold change was calculated and it was denoted NA (not analysed). In cases where expression levels changed from undetectable in 4/4 samples to detectable in 2 or more out of 4, the same statistical test used for the rest of the data could not be applied due to the reduction in sample number. Instead a chi-squared test was performed. This finds that, only when 3 or more of the 4 samples becomes detectable compared with 0/4 in vehicle-treated conditions, will this increase be considered statistically different at the p<0.05. Individual qPCR analysis for genes of particular interest (iNOS, IL-1b, TNF-a and COX-2) all confirmed the gp120-mediated changes identified in the microarray (data not shown). Statistical analysis on gene expression array data was performed using a two-sided Welch’s test on the ∆Ct values against an adjusted p value to limit false positives, using custom-made software designed by Jim Perkins (Bioinformatics Research Group, University College London).

**Supplementary Table 2. List of all gene targets assessed, their Applied Bioscience ID number and the expression changes relative to vehicle control in denatured-gp120_Bal_- and gp120_Bal_-treated BMDM cultures. Genes highlighted bold indicate the housekeeping genes used to normalise the data. *P* values refer to the fold changes in respective treatment groups relative to vehicle-treated.**

|  |  | **Denatured gp120_Bal_** | | | | **gp120_Bal_** | | | |
| --- | --- | --- | --- | --- | --- | --- | --- | --- | --- |
| **ID** | **Target** | **2^-∆∆Ct^** | **(min - max)** | | **p value** | **2^-∆∆Ct^** | **(min - max)** | | **p value** |
| Actb.Rn00667869_m1 | beta-actin | 1 | 0.9 | 1.1 | 0.974 | 1.1 | 1.1 | 1.1 | 0.069 |
| Aif1.Rn00567906_g1 | Aif1 (Iba1) | 0.7 | 0.5 | 1 | 0.88 | 0.8 | 0.6 | 1.1 | 0.709 |
| Alox15.Rn00696151_m1 | Alox15 (Arachidonate 15-lipoxygenase) | 0.8 | 0.3 | 2 | 0.923 | 0.8 | 0.4 | 1.8 | NA |
| Alox5.Rn00563172_m1 | Alox5 (Arachidonate 5-lipoxygenase) | 0.8 | 0.5 | 1.2 | NA | NA | NA | NA | NA |
| Areg.Rn00567471_m1 | Areg (amphiregulin) | NA | NA | NA | NA | NA | NA | NA | NA |
| Artn.Rn01761472_g1 | Artn (artemin) | 0.3 | 0.2 | 0.4 | 0.801 | 0.3 | 0.2 | 0.4 | NA |
| Bdnf.Rn01484928_m1 | BDNF | NA | NA | NA | NA | NA | NA | NA | NA |
| Btc.Rn00673281_m1 | Btc (betacellulin) | NA | NA | NA | NA | NA | NA | NA | NA |
| C3.Rn00566466_m1 | C3 (Complement component 3) | 2.1 | 1.2 | 3.8 | 0.903 | 1.9 | 1 | 3.6 | 0.543 |
| C5.Rn01436156_m1 | C5 (Complement component 5) | NA | NA | NA | NA | NA | NA | NA | NA |
| Ccl1.Rn01752376_m1 | CCL1 | 1.5 | 0.7 | 3.1 | 0.801 | 2 | 0.6 | 6 | NA |
| Ccl11.Rn00569995_m1 | CCL11 | NA | NA | NA | NA | NA | NA | NA | NA |
| Ccl12.Rn01464638_m1 | CCL12 | 12.4 | 0.4 | 374.5 | 0.801 | 1167.2 | 1005 | 1355 | 0.02 |
| Ccl17.Rn00589290_m1 | CCL17 | + | NA | NA | NA | NA | NA | NA | NA |
| Ccl19.Rn01439563_m1 | CCL19 | 0.8 | 0.6 | 1.1 | 0.801 | 14 | 8.7 | 22.5 | NA |
| Ccl2.Rn00580555_m1 | CCL2 | 2.1 | 1 | 4.4 | 0.821 | 134.8 | 107.9 | 168.4 | 0.001 |
| Ccl20.Rn00570287_m1 | CCL20 | 1.7 | 0.7 | 3.8 | NA | 276.6 | 133.9 | 571.6 | 0.0005 |
| Ccl21b.Rn01764651_g1 | CCL21β | NA | NA | NA | NA | NA | NA | NA | NA |
| Ccl22.Rn01536591_m1 | CCL22 | 0.7 | 0.2 | 2.2 | NA | 1.1 | 0.4 | 3.2 | NA |
| Ccl24.Rn01481451_m1 | CCL24 | NA | NA | NA | NA | NA | NA | NA | NA |
| Ccl25.Rn01403351_m1 | CCL25 | NA | NA | NA | NA | NA | NA | NA | NA |
| Ccl26.Rn01481484_m1 | CCL26 | NA | NA | NA | NA | NA | NA | NA | NA |
| Ccl27.Rn01437037_m1 | CCL27 | NA | NA | NA | NA | NA | NA | NA | NA |
| Ccl28.Rn00586715_m1 | CCL28 | NA | NA | NA | NA | NA | NA | NA | NA |
| Ccl3.Rn00564660_m1 | CCL3 | 1.4 | 0.2 | 9 | 0.974 | 127.1 | 53.9 | 299.9 | 0.005 |
| Ccl4.Rn00587826_m1 | CCL4 | 2.5 | 1 | 6.2 | 0.821 | 306.3 | 205.7 | 456.1 | 0.004 |
| Ccl5.Rn00579590_m1 | CCL5 | 0.9 | 0.2 | 3.3 | 0.983 | 474.5 | 342.2 | 657.9 | 0.008 |
| Ccl6.Rn01456402_g1 | CCL6 | 1.1 | 0.6 | 1.9 | 0.974 | 1.3 | 0.5 | 3.2 | 0.792 |
| Ccl7.Rn01467286_m1 | CCL7 | 1.4 | 0.8 | 2.5 | 0.821 | 93.4 | 81.8 | 106.6 | 2.00E-07 |
| Ccl9.Rn01471276_m1 | CCL9 | 1 | 0.4 | 2.2 | 0.974 | 7.5 | 4.3 | 13 | 0.03 |
| Csf1.Rn00576849_m1 | M-CSF1 (macrophage colony stimulating factor 1) | 1.2 | 0.9 | 1.6 | 0.958 | 2.9 | 1.9 | 4.4 | 0.03 |
| Csf2.Rn01456851_m1 | Csf2 | NA | NA | NA | NA | + | NA | NA | NA |
| Csf3.Rn00567344_m1 | Csf3 | NA | NA | NA | NA | + | NA | NA | NA |
| Cx3cl1.Rn00593186_m1 | CX3CL1 | 1.6 | 0.9 | 2.8 | 0.88 | 41.4 | 22.8 | 75.1 | 0.001 |
| Cxcl1.Rn00578225_m1 | CXCL1 | 3.9 | 2.2 | 6.9 | 0.166 | 248 | 198.5 | 309.9 | 2.00E-05 |
| Cxcl10.Rn00594648_m1 | CXCL10 | 5.1 | 2.2 | 11.4 | 0.252 | 939.9 | 737.5 | 1198 | 0.0003 |
| Cxcl11.Rn00788262_g1 | CXCL11 | 5.9 | 1.6 | 22.5 | 0.801 | 3205.5 | 2241.9 | 4583.4 | 3.00E-05 |
| Cxcl12.Rn00573260_m1 | CXCL12 | 1 | 0.6 | 1.6 | 0.974 | 0.9 | 0.5 | 1.8 | 0.884 |
| Cxcl13.Rn01450028_m1 | CXCL13 | 0.9 | 0.5 | 1.7 | NA | 3.2 | 1.2 | 8.2 | NA |
| Cxcl14.Rn01441840_m1 | CXCL14 | 1.2 | 1 | 1.5 | 0.821 | 1 | 0.7 | 1.4 | 0.969 |
| Cxcl16.Rn01496393_m1 | CXCL16 | 1.1 | 0.7 | 1.7 | 0.974 | 2.3 | 1.8 | 2.8 | 0.035 |
| Cxcl17.Rn01764053_m1 | CXCL17 | NA | NA | NA | NA | + | NA | NA | NA |
| Cxcl2.Rn00586403_m1 | CXCL2 | 10 | 3.2 | 31.4 | 0.821 | 2060.5 | 1186.4 | 3578.6 | 0.052 |
| Cxcl3.Rn00593435_m1 | CXCL3 | NA | NA | NA | NA | NA | NA | NA | NA |
| Cxcl6.Rn00573587_g1 | CXCL6 | 2 | 0.8 | 4.7 | 0.88 | 20.6 | 5.2 | 80.9 | 0.04 |
| Cxcl9.Rn00595504_m1 | CXCL9 | 2.1 | 0.9 | 5.2 | 0.801 | 2082.9 | 1525 | 2844 | 3.00E-05 |
| Ebi3.Rn01527778_m1 | Ebi3 (Epstein-Barr virus induced 3) | 0.9 | 0.7 | 1.2 | 0.974 | 1.7 | 1.3 | 2.3 | 0.045 |
| Edn1.Rn00561129_m1 | Edn1 (Endothelin1) | 0.9 | 0.5 | 1.4 | 0.974 | 3.1 | 2 | 4.9 | 0.078 |
| Ereg.Rn00572454_m1 | Ereg (epiregulin) | 1.3 | 0.5 | 3.3 | 0.923 | 6.5 | 3.6 | 11.7 | 0.212 |
| Fgf7.Rn00573319_m1 | Fgf7 (Fibroblast growth factor 7) | 3.9 | 1.9 | 7.9 | 0.974 | 1.3 | 0.6 | 3 | 0.296 |
| Gapdh.Rn99999916_s1 | GAPDH | 1 | 0.8 | 1.3 | 0.974 | 0.9 | 0.8 | 1.1 | 0.758 |
| Hbegf.Rn00564075_m1 | Hbegf (Heparin-binding EGF-like growth factor) | 0.9 | 0.3 | 3 | 0.974 | 1.8 | 1.3 | 2.3 | 0.445 |
| Hprt1.Rn01527840_m1 | Hprt1 (hypoxanthine phosphoribosyl transferase 1) | 3.6 | 0.2 | 69.3 | 0.903 | 0.8 | 0.6 | 1 | 0.334 |
| Ifng.Rn00594078_m1 | IFNγ | NA | NA | NA | NA | NA | NA | NA | NA |
| Il10.Rn01644839_m1 | IL-10 | 1 | 0.7 | 1.3 | 0.983 | 2.3 | 0.6 | 9.2 | NA |
| Il11.Rn00591721_m1 | IL-11 | 0 | NA | NA | NA | NA | NA | NA | NA |
| Il12a.Rn00584538_m1 | IL-12α | NA | NA | NA | NA | 4.6 | 1.1 | 19.5 | 0.297 |
| Il12b.Rn00575112_m1 | IL-12β | NA | NA | NA | NA | + | NA | NA | NA |
| Il13.Rn00587615_m1 | IL-13 | NA | NA | NA | NA | NA | NA | NA | NA |
| Il15.Rn00689964_m1 | IL-15 | 1.4 | 0.7 | 2.7 | 0.801 | 43.1 | 30.1 | 61.7 | 0.003 |
| Il16.Rn01477722_m1 | IL-16 | 0 | NA | NA | NA | - | NA | NA | NA |
| Il18.Rn01422083_m1 | IL-18 | 1.3 | 0.6 | 2.6 | 0.974 | 2.9 | 1.9 | 4.3 | 0.145 |
| Il19.Rn01490483_m1 | IL-19 | NA | NA | NA | NA | NA | NA | NA | NA |
| Il1a.Rn00566700_m1 | IL-1α | 8.9 | 3.2 | 25.2 | 0.503 | 2016.3 | 1336 | 3043 | 0.0004 |
| Il1b.Rn00580432_m1 | IL-1β | 8.5 | 5.4 | 13.6 | 0.155 | 948.3 | 798.4 | 1126 | 0.001 |
| Il2.Rn99999181_m1 | IL-2 | NA | NA | NA | NA | NA | NA | NA | NA |
| Il20.Rn01483978_m1 | IL-20 | NA | NA | NA | NA | - | NA | NA | NA |
| Il21.Rn01755623_m1 | IL-21 | NA | NA | NA | NA | NA | NA | NA | NA |
| Il23a.Rn00590334_g1 | IL-23α | NA | NA | NA | NA | + | NA | NA | NA |
| Il24.Rn00591159_m1 | IL-24 | NA | NA | NA | NA | NA | NA | NA | NA |
| Il27.Rn01510484_m1 | IL-27 | 6.5 | 3.7 | 11.2 | 0.821 | 2446 | 1857.3 | 3221.5 | 0.026 |
| Il3.Rn00580435_m1 | IL-3 | NA | NA | NA | NA | + | NA | NA | NA |
| Il33.Rn01759837_m1 | IL-33 | 0.5 | 0.3 | 1 | 0.821 | 1.2 | 1 | 1.4 | 0.792 |
| Il4.Rn01456866_m1 | IL-4 | NA | NA | NA | NA | + | NA | NA | NA |
| Il5.Rn01459975_m1 | IL-5 | NA | NA | NA | NA | + | NA | NA | NA |
| Il6.Rn00561420_m1 | IL-6 | 1.3 | 0.1 | 23.5 | 0.983 | 18.6 | 1.3 | 266 | NA |
| Il7.Rn00681900_m1 | IL-7 | 1.2 | 0.5 | 3.2 | 0.923 | 0.5 | 0.3 | 0.8 | NA |
| Il9.Rn01448718_m1 | IL-9 | NA | NA | NA | NA | NA | NA | NA | NA |
| Kitlg.Rn00442977_m1 | Kitlg (KIT ligand, MCF, SCF stem cell factor) | 0.8 | 0.2 | 2.9 | 0.974 | 0.2 | 0.1 | 0.5 | 0.073 |
| Lif.Rn00573491_g1 | LIF | 1.4 | 0.3 | 5.9 | 0.923 | 23 | 21.6 | 24.5 | 0.095 |
| LOC301289.Rn01757168_m1 | LOC301289 (similar to IL-17 precursor (CTLA-8)) | NA | NA | NA | NA | + | NA | NA | NA |
| Lta4h.Rn01503878_m1 | Lta4h (Leukotriene A4 hydrolase) | 1.2 | 0.9 | 1.6 | 0.821 | 1 | 0.8 | 1.4 | 0.884 |
| Mif.Rn00821234_g1 | MIF (macrophage migration inhibitory factor) | 1 | 0.9 | 1.1 | 0.974 | 0.7 | 0.6 | 0.8 | 0.138 |
| Ngf.Rn01533872_m1 | NGF | 1.1 | 0.7 | 1.6 | 0.983 | 2 | 0.6 | 7 | NA |
| Nos2.Rn00561646_m1 | iNOS | 2 | 0.7 | 5.7 | 0.801 | 2244 | 1846 | 2728 | 0.0002 |
| Nrg1.Rn00580917_m1 | NRG1 (neuregulin 1) | 0.7 | 0.2 | 2 | 0.923 | 0.2 | 0.2 | 0.4 | NA |
| Nrg1.Rn01482165_m1 | NRG1 (neuregulin 1) | 0 | NA | NA | NA | 0 | 0 | 0 | NA |
| Pf4.Rn01768298_g1 | Pf4 (CXCL4, platelet factor 4) | 1 | 0.8 | 1.3 | 0.974 | 0.8 | 0.6 | 1.1 | 0.553 |
| Ppbp.Rn00596603_g1 | Ppbp (CXCL7, pro-platelet basic protein) | NA | NA | NA | NA | - | NA | NA | NA |
| Prok2.Rn01421308_m1 | Prok2 (Prokineticin) | NA | NA | NA | NA | - | NA | NA | NA |
| Ptges.Rn00572047_m1 | Ptges (prostaglandin E synthase) | 1.1 | 0.2 | 6.4 | 0.983 | 46.8 | 15.4 | 142.2 | 0.02 |
| Ptgs2.Rn01483828_m1 | Ptgs2 (COX2, prostaglandin-endoperoxide synthase 2) | 0.4 | 0.2 | 1.2 | 0.923 | 57.2 | 43.1 | 76 | 0.307 |
| Tnf.Rn99999017_m1 | TNF-α | 2.4 | 0.7 | 8.6 | 0.821 | 313.7 | 199.1 | 494.3 | 2.00E-05 |
| Txlna.Rn01516136_m1 | Txlnα (α-Taxilin) | 0.9 | 0.7 | 1 | 0.821 | 0.5 | 0.3 | 0.9 | 0.145 |
| X18S.Hs99999901_s1 | X18S (Eukaryotic 18S rRNA) | 1 | 0.9 | 1.2 | 0.974 | 1 | 0.9 | 1.1 | 0.803 |
| Xcl1.Rn00592605_m1 | XCL1 (Lymphotactin) | 1.9 | 0.4 | 8.9 | 0.801 | 5.2 | 0.6 | 43.2 | 0.351 |

**2.2) Staining of DRG cultures with the macrophage marker ED1**


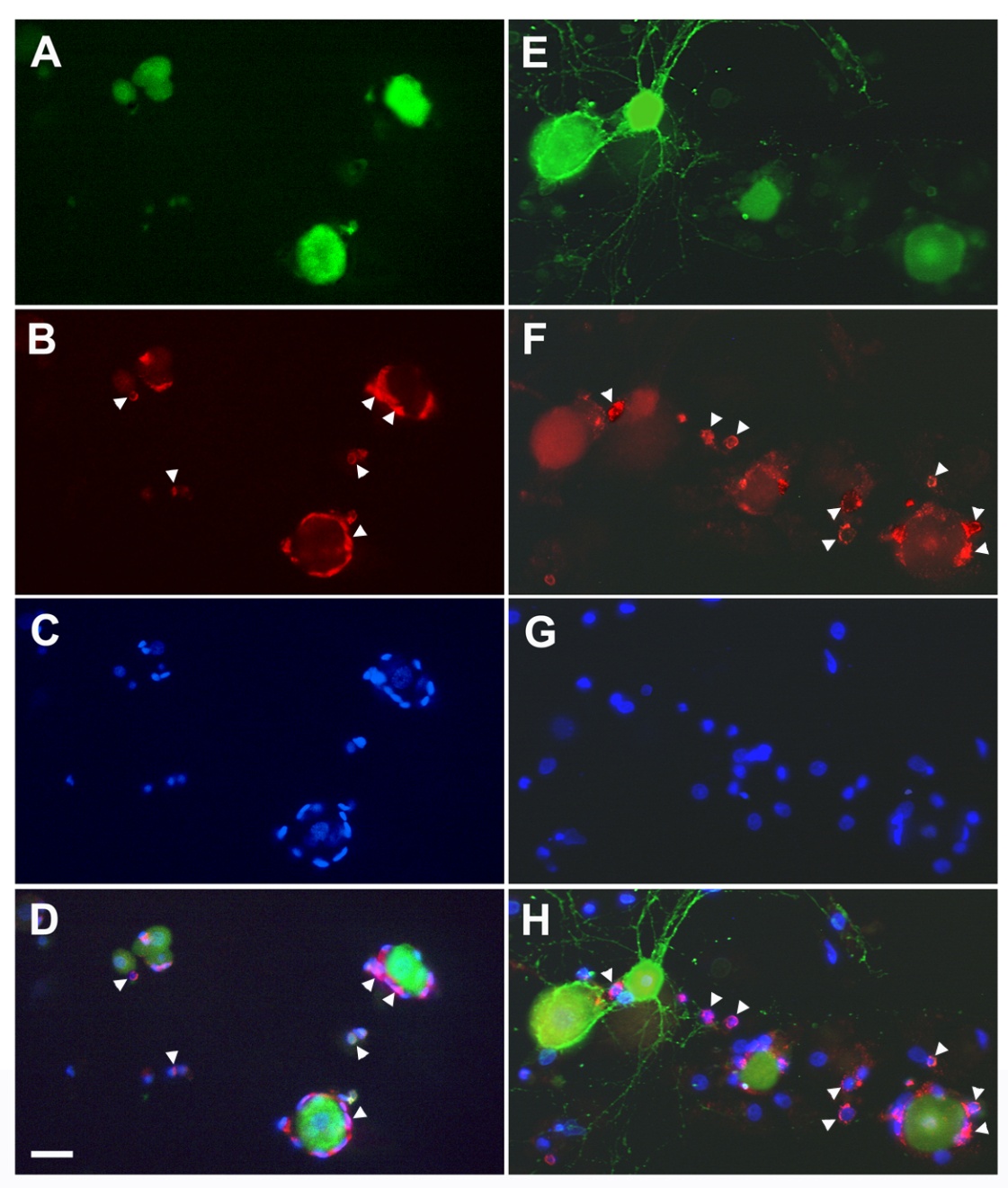


Supplementary Fig. 3. ED1 staining of primary adult rat mixed DRG neuronal cultures. A–D were taken from DRG cultures at 2.5h following cell plating. E–H were taken from DRG cultures at 1 DIV (days *in vitro*). GAP-43 (green, A and E) was used as a marker for DRG neurones; ED1 (red, B and F) was used as a marker for macrophages; and Hoechst (blue, C and G) was used to label the nuclei of cells. D and H are overlays of A–C and E–G, respectively. Representative examples of ED1-positive cells surrounding or in between DRG neurones are shown by arrowheads, at both time points. Scale bar=25µm.

**2.3) Intradermal injection of gp120 induces release of pro-inflammatory cytokines**

The expression of five key cytokines (iNOS, TNF-α, CCL2, IL-1β, and IL-6) identified to be upregulated in the qPCR array, were also shown to be significantly upregulated in the intraplantar paw 5hr after an injection with 400ng gp120_Bal_ (Supplementary Table 3). This confirmed that the BMDM *in vitro* experimental set-up, and the results of the cytokine array, are biologically relevant and reproducible *in vivo*. The observed fold changes were lower *in vivo* than those identified in the microarray, however this likely reflects the heterogeneity of cells collected in an *in vivo* set-up.

|  | **Fold difference relative to naїve (mean 2^-∆∆CT^ [SD])** | | |
| --- | --- | --- | --- |
| **Gene target** | **Naїve** | **Den.gp120_Bal_** | **gp120_Bal_** |
| **iNOS** | 2.4 [2.7] | 3.5 [1.7] | 49.2 [28.4] * |
| **TNF-α** | 1.1 [0.6] | 0.7 [0.4] | 7.4 [0.9] *** |
| **CCL2** | 1.5 [1.7] | 1.2 [0.7] | 16.1 [14.7] * |
| **IL-1β** | 1.0 [0.3] | 2.1 [1.1] | 11.5 [5.2] *** |
| **IL-6** | 7.3 [13.6] | 4.5 [4.4] | 88.8 [47.6] ** |

**Supplementary Table 3. The fold changes of key pro-inflammatory cytokines (relative to sham) in ipsilateral epidermal plantar tissue.** Data presented as mean [SD]. *p<0.05, **p<0.01; ***p< 0.003; n=4.

**2.4) Modulation of macrophage phenotype by IL-4**


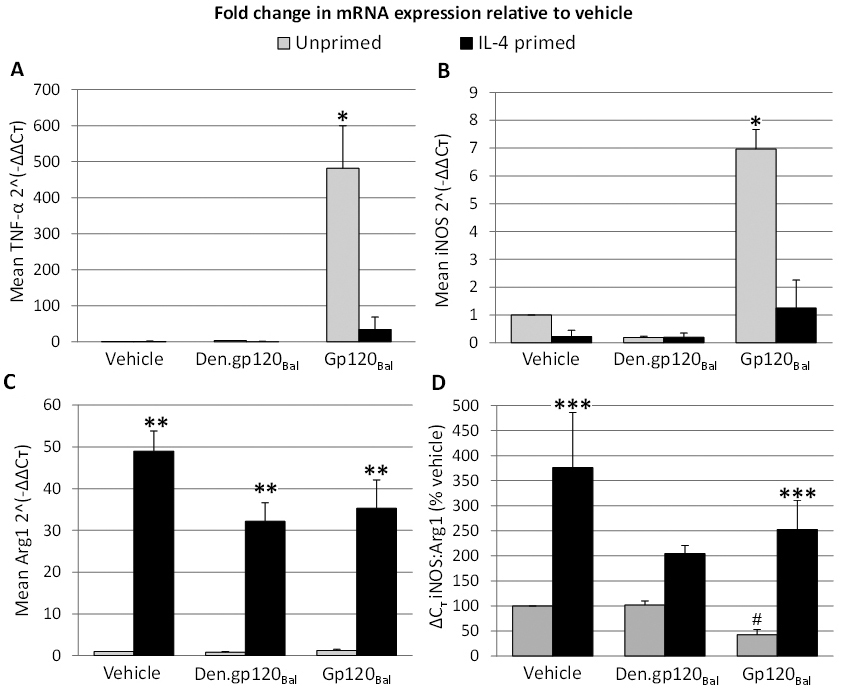


Supplementary Fig. 4. Effect of IL-4 priming on TNF-α, iNOS, and Arg1 mRNA expression following exposure to gp120. Primary BMDM cells were serum-deprived either in the absence or presence of IL-4 (5ng ml^-1^) for 24 hs prior to stimulation for 4h with vehicle, 2nM denatured gp120_Bal_, or 2nM gp120_Bal_ in IMDM. (A-C) mRNA expression changes of TNF-α (n=3/treatment/priming condition), iNOS (n=4/treatment/priming condition), and Arg1 (arginase 1; n=4/treatment/priming condition) in unprimed and IL-4-primed BMDMs following treatment relative to the expression levels in unprimed vehicle-treated BMDMs (n=4). (D) The ∆C_T_ iNOS:Arg1 ratio was quantified, for each treatment in both unprimed and IL-4-primed BMDMs, from their relative qPCR ∆C_T_ values and presented as a percentage of the ∆C_T_ iNOS:Arg1 ratio in unprimed vehicle-treated BMDMs. Data presented as mean 2^-∆∆Ct^ fold change ± SEM or as a percentage of vehicle mean 2^-∆∆Ct^ (n=4, qPCRs performed in duplicate for each sample). *p<0.05 vs. vehicle or denatured gp120 (both primed and unprimed), and gp120 (primed); **p<0.05 vs. unprimed within each treatment; ***p<0.05 vs. unprimed within each treatment; #p<0.05 vs. unprimed denatured gp120; using two-way ANOVA and Tukey’s post-hoc analysis.

**2.5) Modulation of macrophage phenotype by EPA**


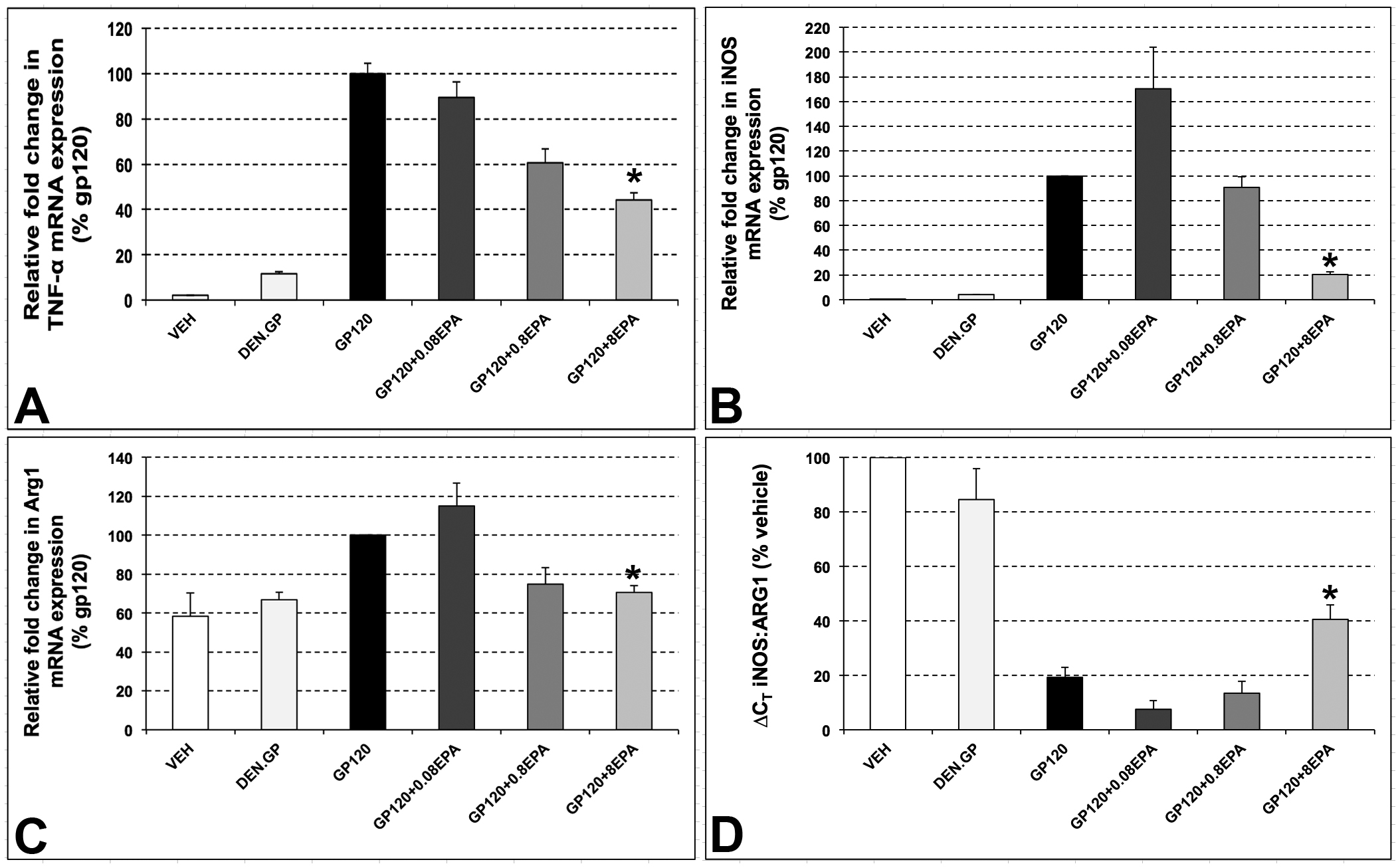


Supplementary Fig. 5. Effect of EPA on TNF-α, iNOS, and Arg1 mRNA expression following exposure to gp120. Primary BMDM cells were treated with vehicle, 2nM denatured gp120_Bal_, 2nM gp120_Bal_ in IMDM, in the absense or presense of 0.08μM, 0.8μM, or 8μM EPA for 4h (n=3 per treatment). (A-C) fold changes in mRNA expression of TNF-α, iNOS and Arg1 in BMDMs following each treatment relative to the expression levels in BMDMs treated with gp120_Bal_ in vehicle. (D) The ∆C_T_ iNOS:Arg1 ratio was quantified, for each treatment in BMDMs, from their relative qPCR ∆C_T_ values and presented as a percentage of the ∆C_T_ iNOS:Arg1 ratio in vehicle-treated BMDMs. Data presented as a percentage of gp120_Bal_ mean 2^-∆∆Ct^ fold change (SEM) (for TNF-α, iNOS, and Arg1 mRNA expression) or as a percentage of vehicle mean 2^-∆∆Ct^ (for ∆C_T_ iNOS:Arg1 ratio). *p<0.05 vs. gp120 and gp120 with 0.08μM EPA; using two-way ANOVA and Tukey’s post-hoc analysis.

**3. Additional discussion**

3.1) Reasons why it took 24h for gp120 neurotoxicity to emerge

Neuronal outgrowth of gp120-treated cultures was not slowed during the first 16h of gp120 exposure, as we observed a similar growth in gp120-treated cultures when compared to that of the control cultures; but a strong retraction of established neurites had occurred after 24h. This mirrors the ‘dying-back’ pattern of axonal degeneration of established neurones observed in the clinical setting and also in SIV-infected macaques.[^9^](#_ENREF_9) The effect of gp120 on neurites would not be expected to be instantaneous, especially when hypothesising the macrophage as essential intermediary cells. The biochemical and anatomical processes take time.

3.2) Reasons why we only looked at changes up to 48h

While it would be interesting to assess whether neurones recover from an acute gp120 insult, this was not the purpose of these experiments. The focus of our studies was to identify the mechanisms and cells involved in the establishment of neurite degeneration. As significant neurite retraction had occurred by 24h (Fig. 1J), and was stable up until 48h, it was not within the remit of our studies to assess later time points. To study this requires a different experimental design. However, it would be interesting to study later time points to determine whether neurons are permanently degenerated, even once the gp120 input is removed, or whether they regain their regenerative ability.

3.3) Reasons why we only focused on TNF-α in this study and the importance of cytokine synergism

TNF-α is one of the most prominently induced genes following macrophage activation, making it a useful and consistent biomarker to clarify the acute responses of BMDMs to gp120. Furthermore, TNF-α induces the expression of numerous cellular genes through activation of NF-κB.[^10^](#_ENREF_10) Certain TNF-related haplotypes have been identified as a risk factor for developing HIV-SN.[^11^](#_ENREF_11) It has been implicated both in neuronal degeneration and the development and maintenance of neuropathic pain in animal and clinical studies, and may be a key factor released from gp120_Bal_-treated BMDM. TNF-α is an important cytokine involved in the early phase of immune activation; functioning as a potent chemoattractant to recruit additional immune cells to the site of injury. While we only tested TNF-α, chosen due to its highlighted association with development of HIV-SN and our own work, it is unlikely to be the sole mediator involved.

Here, we demonstrated that 2nM TNF-α, a concentration consistent with the clinical setting, induced significant neurite outgrowth reduction in DRG neurones. We cannot exclude possible synergistic interactions among other potential mediators released by gp120-exposed BMDMs. For example, PG release is driven through the switching on and off of the COX pathway by NO and NO donors.[^16^](#_ENREF_16) COX-2 activity can be enhanced through direct iNOS binding.[^17^](#_ENREF_17) Considering that these genes were also potently upregulated suggests they could be released jointly and with the cross talk in their modulation, it is conceivable that synergy between the actions of PG and NO exacerbates neuronal damage. Future studies are needed to further clarify the fascinating interplay between immunity and neurotoxicity and the relevant involvement in HIV-SN.

3.4) Causes for the migration of circulating macrophages to the peripheral nervous system

Although we have established a dominant role of peripheral macrophages in the establishment of gp120-associated neurotoxicity it is still to be determined the mechanism by which macrophages come to surround peripheral neurons. As well as exciting macrophages, gp120 is a chemokine and therefore a chemoattractant to immune cells including the circulating macrophages. Furthermore, our previous data have shown that macrophages accumulate at the site of application of gp120,[^18^](#_ENREF_18) which is consistent with the clinical setting that shows a positive correlation between the degree of macrophage infiltration and the level of neuronal degeneration. An alternative view is that gp120 induces gene expression changes directly in neurons via activation of neuronally-expressed CCR5 or CXCR4.[^19^](#_ENREF_19) These changes may not directly induce neurite degeneration but rather the release of unknown chemoattractant mediators, which then recruit immune cells and the subsequent cascade of activation ensues. It is also possible that in the clinical setting where a patient has been treated with relevant antiretroviral drugs the initial injury signal may arise in association from a “damaged peripheral nerve” as a result of antiretroviral drug-induced neurotoxicity. Further studies are needed, to map the complete temporal course of gp120-associated neurotoxicity, with consideration for all cell types involved.

3.5) The effects of IL-4 and EPA on macrophage phenotypes

Having seen that BMDM polarisation state can alter to an M1 state following exposure to gp120 to crate this event, we have become interested in whether macrophage responses can be modulated before this amplification occurs. Our preliminary results show have that both priming BMDMs into an M2 state with IL-4, and co-treatment with the omega-3 fatty acid eicosapentaenoic acid, significantly attenuate gp120-induced TNF-α mRNA expression (Supplementary Fig. 4 and Fig. 5). We propose that modulating this macrophage immune response, could offer greater opportunity for clinical intervention against HIV-associated neuropathy, over targeting a single entity later in the pathway. Our future studies aim to explore this fascinating prospect further and identify whether similar gains could be achieved with neuropathic pain.

3.6) Cell death and gp120 neurotoxicity

We only assessed cell death in our neuronal cultures that were exposed to direct application of TNF-α versus a vehicle control. No significant difference was found, when the numbers of fragmented nuclei were compared. Until recently, it was believed that axonal degeneration and apoptosis were mechanistically linked and directly connected, however recent studies have suggested otherwise. Three factors have brought researchers to this conclusion: 1) it has been shown that caspase-3, of the family of cysteine proteases normally indicative of apoptosis in neurons,[^21^](#_ENREF_21) is not activated in the axons during axonal degeneration;[^22^](#_ENREF_22) 2) caspase inhibitors prevent the cell death associated with NGF deprivation but not axonal degeneration;[^22^](#_ENREF_22) and 3) the gene for slow Wallerian degeneration protects against axonal degeneration but not cell apoptosis following insult.[^23^](#_ENREF_23) Together these data provide evidence that axonal degeneration and apoptosis are mechanistically distinct therefore when understanding the pathology that drives HIV-SN it is important to consider cell death and axonal degeneration independently. For this reason, quantifying neuronal cell death did not form a primary part of our studies that were more focused on identifying the factors and cells involved in initiating that degenerative process. However, we do fully appreciate that cell death will have an important clinical impact on patients with HIV infection.

3.7) Glutamate and gp120 neurotoxicity

Previous studies have shown that the neurotoxicity of activated macrophages is mediated via glutamate release.[^24^](#_ENREF_24) From our own work, we have shown the involvement of quinolinic acid, which is in line with the previous findings,[^24^](#_ENREF_24) showing that excitatory mediators identified to be upregulated in different *in vitro* macrophage models after gp120 treatment include glutamate and quinolinic acid. Activated macrophages produce a vast majority of quinolinic acid, the excitotoxic metabolite of L-tryptophan and endogenous agonist of NMDA receptors and CSF concentrations of quinolinic acid have been significantly correlated to the severity of the neuropsychological deficits associated with HIV-1 infection.[^25^](#_ENREF_25) Synergism exists between quinolinic acid and glutamate that can lead to glutamate neurotoxicity.[^26^](#_ENREF_26) These established excitotoxic effects and magnitude of increases in CSF raise the possibility that quinolinic acid and glutamate may both play a key role in the pathogenesis of neuronal dysfunction associated with HIV-1 infection.

3.8) Confirming that our genes of special interest were also upregulated in plantar tissue 5hr following intradermal injection of gp120_Bal_ gave biological validation to our qPCR array data (Supplementary Table 3).

Reference

1. Abdulla EM, Campbell IC. Use of neurite outgrowth as an in vitro method of assessing neurotoxicity. *Annals of the New York Academy of Sciences* 1993; 679: 276-9
2. Ronaldson PT, Bendayan R. HIV-1 viral envelope glycoprotein gp120 triggers an inflammatory response in cultured rat astrocytes and regulates the functional expression of P-glycoprotein. *Molecular pharmacology* 2006; 70: 1087-98
3. Thacker MA, Clark AK, Bishop T, et al. CCL2 is a key mediator of microglia activation in neuropathic pain states. *Eur J Pain* 2009; 13: 263-72
4. Invitrogen: http://tools.invitrogen.com/content.cfm?pageid=9716.
5. Peinnequin A, Mouret C, Birot O, et al. Rat pro-inflammatory cytokine and cytokine related mRNA quantification by real-time polymerase chain reaction using SYBR green. *BMC immunology* 2004; 5: 3
6. Sousa CT, Brito TS, Lima FJ, et al. Sildenafil decreases rat tracheal hyperresponsiveness to carbachol and changes canonical transient receptor potential gene expression after antigen challenge. *Brazilian journal of medical and biological research = Revista brasileira de pesquisas medicas e biologicas / Sociedade Brasileira de Biofisica [et al]* 2011; 44: 562-72
7. Pardo CA, McArthur JC, Griffin JW. HIV neuropathy: insights in the pathology of HIV peripheral nerve disease. *J Peripher Nerv Syst* 2001; 6: 21-7
8. Polydefkis M, Yiannoutsos CT, Cohen BA, et al. Reduced intraepidermal nerve fiber density in HIV-associated sensory neuropathy. *Neurology* 2002; 58: 115-9
9. Laast VA, Shim B, Johanek LM, et al. Macrophage-mediated dorsal root ganglion damage precedes altered nerve conduction in SIV-infected macaques. *Am J Pathol* 2011; 179: 2337-45
10. Lee C, Tomkowicz B, Freedman BD, Collman RG. HIV-1 gp120-induced TNF-α production by primary human macrophages is mediated by phosphatidylinositol-3 (PI-3) kinase and mitogen-activated protein (MAP) kinase pathways. *Journal of Leukocyte Biology* 2005; 78: 1016-23
11. Cherry CL, Kamerman PR, Bennett DLH, Rice ASC. HIV-associated sensory neuropathy: still a problem in the post-stavudine era? *Future Virology* 2012; 7: 840-54
12. Gao YJ, Ji RR. Chemokines, neuronal-glial interactions, and central processing of neuropathic pain. *Pharmacology & therapeutics* 2010; 126: 56-68
13. McMahon SB, Cafferty WB, Marchand F. Immune and glial cell factors as pain mediators and modulators. *Experimental neurology* 2005; 192: 444-62
14. White FA, Bhangoo SK, Miller RJ. Chemokines: integrators of pain and inflammation. *Nature reviews Drug discovery* 2005; 4: 834-44
15. Zelenka M, Schafers M, Sommer C. Intraneural injection of interleukin-1beta and tumor necrosis factor-alpha into rat sciatic nerve at physiological doses induces signs of neuropathic pain. *Pain* 2005; 116: 257-63
16. Mollace V, Muscoli C, Masini E, Cuzzocrea S, Salvemini D. Modulation of prostaglandin biosynthesis by nitric oxide and nitric oxide donors. *Pharmacological reviews* 2005; 57: 217-52
17. Kim SF, Huri DA, Snyder SH. Inducible nitric oxide synthase binds, S-nitrosylates, and activates cyclooxygenase-2. *Science* 2005; 310: 1966-70
18. Wallace VCJ, Blackbeard J, Pheby T, et al. Pharmacological, behavioural and mechanistic analysis of HIV-1 gp120 induced painful neuropathy. *Pain* 2007; 133: 47-63
19. Oh SB, Tran PB, Gillard SE, Hurley RW, Hammond DL, Miller RJ. Chemokines and Glycoprotein120 Produce Pain Hypersensitivity by Directly Exciting Primary Nociceptive Neurons. *The Journal of neuroscience : the official journal of the Society for Neuroscience* 2001; 21: 5027-35
20. Huang W, Calvo M, Karu K, et al. A clinically relevant rodent model of the HIV antiretroviral drug stavudine induced painful peripheral neuropathy. *Pain* 2013
21. Porter AG, Janicke RU. Emerging roles of caspase-3 in apoptosis. *Cell death and differentiation* 1999; 6: 99-104
22. Finn JT, Weil M, Archer F, Siman R, Srinivasan A, Raff MC. Evidence that Wallerian degeneration and localized axon degeneration induced by local neurotrophin deprivation do not involve caspases. *The Journal of neuroscience : the official journal of the Society for Neuroscience* 2000; 20: 1333-41
23. Lunn ER, Perry VH, Brown MC, Rosen H, Gordon S. Absence of Wallerian Degeneration does not Hinder Regeneration in Peripheral Nerve. *The European journal of neuroscience* 1989; 1: 27-33
24. Yawata I, Takeuchi H, Doi Y, Liang J, Mizuno T, Suzumura A. Macrophage-induced neurotoxicity is mediated by glutamate and attenuated by glutaminase inhibitors and gap junction inhibitors. *Life sciences* 2008; 82: 1111-6
25. Heyes MP, Brew BJ, Martin A, et al. Quinolinic acid in cerebrospinal fluid and serum in HIV-1 infection: relationship to clinical and neurological status. *Annals of neurology* 1991; 29: 202-9
26. Guillemin GJ, Croitoru-Lamoury J, Dormont D, Armati PJ, Brew BJ. Quinolinic acid upregulates chemokine production and chemokine receptor expression in astrocytes. *Glia* 2003; 41: 371-81
